# Leucine to proline substitution by SNP at position 197 in Caspase-9 gene expression leads to neuroblastoma: a bioinformatics analysis

**DOI:** 10.1007/s13205-012-0088-y

**Published:** 2012-09-18

**Authors:** Arpita Kundu, Susmita Bag, Sudha Ramaiah, Anand Anbarasu

**Affiliations:** Medical and Biological Computing Laboratory, School of Biosciences and Technology, VIT University, Vellore, 632014 Tamil Nadu India

**Keywords:** CASP9, Leucine, Neuroblastoma, Proline, rs1052574

## Abstract

To understand the role of CASP9 (Caspase-9) gene products in relation to neuroblastoma disease, we have analyzed the single nucleotide polymorphisms (SNPs) associated with this gene. This can help us understand the genetic variations that can alter the function of the gene products. A total of 941 SNPs are investigated for CASP9 gene. To determine whether a non-synonymous SNP (nsSNP) in this gene affects its protein product, we used certain computational tools which predicted one nsSNP, rs1052574, to have deleterious phenotypic effect. This polymorphic variant results in amino acid substitution from leucine to proline at 197 position, i.e., from acyclic amino acid to a 5-membered amino acid which resides in the buried area of the protein with a high level of conservation. This amino acid substitution shows a transition from helix to coil in the mutant protein. Hence, due to the complete alteration in the structural property of the amino acid side chain, the stability of the protein is reduced which may affect the function of CASP9 protein, leading to deregulation of apoptosis and neuroblastoma development.

**Electronic supplementary material:**

The online version of this article (doi:10.1007/s13205-012-0088-y) contains supplementary material, which is available to authorized users.

## Introduction

Neuroblastoma (NB) is the most common extracranial tumor of childhood, arising from neural crest cells, accounting for approximately 10 % of pediatric cancers (Gale et al. 1982; Brodeur et al. 1992; Schor 1999). NB is characterized by diverse behavior ranging from rapid malignant progression to spontaneous regression (Lastowska et al. 2001). It has been suggested that genetic susceptibility to NB is now highly probable (Shojaei-Brosseau et al. 2004). Attention has been focused on determining the specific genetic alterations in tumors affecting the prognosis and leading to targeted therapies for the individual cancer patient (Heinrichs and Look 2007). The most common form of genetic variation in the human genome are single nucleotide polymorphisms (SNPs), accounting for heritable inter-individual variability in complex phenotypes (Liaoa and Lee 2010; Suh and Vijg 2005). Several biological markers related to the outcome of NB disease have been identified (Cattelani et al. 2008). Apoptosis, the process of cell elimination play a vital role in maintaining cellular homeostasis, cell proliferation and differentiation. Disturbances in the cell death process may lead to uncontrolled cell growth and tumor formation (Zhivotovsky and Orrenius 2006). It has been proposed that aberrations in apoptosis contribute to NB progression (Abel et al. 2005). Caspase-9 (CASP9) gene, a key regulator of the apoptotic signaling system is mapped to the consensus region deleted in all NB cases with 1p deletion (Ohira et al. 2000). Thus, it is considered to be a good candidate gene for NB (Abel et al. 2002). Recent evidence suggests that CASP9, a critical member of the mitochondrial-mediated apoptotic protease cascade, is expressed to a low extent in tumors of NB patients, suggesting that dysregulation of apoptosis is likely to be instrumental in the development or progression of childhood tumor neuroblastoma (Abel et al. 2002, 2005). Single nucleotide changes in CASP9 gene, leading to the reduced expression of the protein, have been studied in NB tumors (Abel et al. 2002). Bioinformatics tools used to screen the potentially deleterious SNPs based on the gene of interest have been documented (Mah et al. 2011). Attention has been focused on non-synonymous SNPs (nsSNPs) for an association study of genetic diseases which can be useful to examine the potential impact of an amino acid variant on the function of the encoded protein (Johnson et al. 2005). The substitution of one amino acid for another generally results in conformational changes in the immediate vicinity of the substituted site which leads to a significant alteration in thermodynamic stability of the single mutant site from that of the corresponding native protein (Shortle and Sondek 1995; Querol et al. 1996). A computational approach has also been employed to study the effect of the protein stability upon mutation (Guerois et al. 2002). Hydrogen bonding, being one of the major structural determinants in protein molecules, helps us understand protein structure and motions. It also contributes to the specificity of intramolecular interactions in biological systems (Kortemmea et al. 2003). Such type of interactions can be affected by any amino acid variant in the protein molecule due to mutation (Wang and Moult 2001). Thus, to analyze the intramolecular interactions upon mutations, we have carried out a computational analysis of the hydrogen bonds (H-bonds) across the modeled protein molecules.

We assume that there are no bioinformatics approaches to document the decreased expression of mutated CASP9 gene. This prompted us to carry out the analysis of phenotypic impact of nsSNPs of CASP9 gene and their effect on structural stability of the mutated protein. Our results will provide an insight to the researchers for understanding the regulatory role played by CASP9 in apoptosis and the genetic consequence of NB.

## Materials and methods

### Data source

The SNPs associated with CASP9 gene were obtained from the single nucleotide polymorphism database (dbSNP) (Wheeler et al. 2008) and the reference SNP (rs) IDs are listed in the Supplementary table. There was a total of 941 SNPs associated with CASP9.

### F-SNP identifies nsSNPs by mining Ensembl database

For selecting nsSNPs of CASP9 gene, we used functional single nucleotide polymorphism (F-SNP) database (Lee and Shatkay 2008). The F-SNP database identified nsSNPs that had deleterious effects on protein structure or function, or impede post-translational modification (Lee and Shatkay 2008). The Ensembl database (Hubbard et al. 2009; Ramensky et al. 2002) was mined to identify nsSNPs. Ensembl provided the number of synonymous and non-synonymous SNPs related to the gene of interest. Each gene had an associated GeneSNPView page which showed SNP locations and effects related to gene and protein structures (Reumers et al. 2005).

### Functional effects of nsSNPs are assessed using F-SNP database

We used F-SNP database which provided a comprehensive collection of functional information about SNPs with respect to four biomolecular functional categories: protein coding, splicing regulation, transcriptional regulation and post-translation effects. To obtain the functional effects of SNPs, it made use of large variety of publicly available tools and resources (Lee and Shatkay 2008).

### Mining different tools by F-SNP to predict the functional effects of nsSNPs in protein coding region

The tools that were utilized for identifying the coding nsSNPs include Sorting Intolerant from Tolerant (SIFT) (Ng and Henikoff 2003; Shen et al. 2006), Polymorphism Phenotyping (PolyPhen) (Johnson et al. 2005; Zhu et al. 2008), SNPeffect (Reumers et al. 2005, 2006), large-scale annotation of coding nsSNPs (LS-SNP) (Karchin et al. 2005; Ryan et al. 2009) and SNPs3D (Yue et al. 2006).

### Identifying changes in the splicing regulation system by F-SNP

F-SNP database mined certain computational prediction tools that were developed for locating splicing elements and identifying the exon/intron structures of genes (Cartegni et al. 2003). Exonic splicing enhancer (ESEfinder) (Cartegni et al. 2003), relative enhancer and silencer classification by unanimous enrichment (RESCUE-ESE) (Fairbrother et al. 2004), exonic splicing regulator (ESRSearch) (Fairbrother et al. 2002) and putative exonic splicing enhancer (PESE) (Zhang et al. 2005) were used for locating SNPs in exonic-splice regions.

### F-SNP mines different tools to examine transcriptional regulation and post-translational modification sites

Golden Path was mined to identify SNPs in transcriptional regulatory regions (Kuhn et al. 2007). OGPET (Gerken et al. 2004) was used to examine post-translational modification sites. OGPET predicted the O-glycosylation sites (Lanver et al. 2010).

### Predicting the phenotypic effect of nsSNPs

We employed the nsSNPAnalyzer (Bao et al. 2005) to predict nsSNP’s phenotypic effect (disease associated vs. neutral). nsSNPAnalyzer annotated the structural environment of each SNP site using the ENVIRONMENT program (Bowie et al. 1991). The program combined three parameters: (i) area of the side chain which was classified as buried, partially buried and exposed according to its solvent accessibility. The buried class was subdivided into three classes (B1, B2 and B3) in order of increasing environmental polarity. The partially buried class was subdivided into P1 and P2 in order of increasing polarity. The exposed side chain was labeled as E; (ii) fraction polar score, giving a measure of environmental polarity related to hydrogen bond formation; and (iii) secondary structure (helix, sheet or coil). The server also integrated evolutionary information and the normalized probability of the substitution was calculated using SIFT program (Ng and Henikoff 2003). nsSNPAnalyzer then used a machine learning method, Random Forests (Bao et al. 2005), to predict the phenotypic class of nsSNPs (Bao and Cui 2005).

### Identifying highly conserved positions in protein sequence

The ConSurf server was used for calculating the evolutionary conservation of amino acid positions in proteins using an empirical Bayesian inference (Ashkenazy et al. 2010). It automated the algorithmic tools to identify the functionally important regions in query proteins by surface mapping of the level of conservation of the amino acid sites among their close sequence homologs (Glaser et al. 2003). The conservation scores were divided into a discrete scale of nine grades for visualization, from the most variable positions (grade 1), through intermediately conserved positions (grade 5), to the most conserved positions (grade 9) (Ashkenazy et al. 2010).

### Comparative modeling of wild and mutant CASP9 proteins

Modeller 9.10, comparative modeling software was executed to build protein models from the templates obtained from sequence similarity with the target protein sequence (Fisher and Sali 2003). The prediction process consisted of target-template alignment, model building and model evaluation (Eswar et al. 2007).

### Analyzing the structural effect of protein upon mutation

SNPeffect server (Reumers et al. 2005) was employed to analyze the structural effect of the nsSNP. The server utilized the force-field FoldX (Guerois et al. 2002) to evaluate the protein stability upon mutation. FoldX program estimated the mutational free energy change on the stability of a protein (Schymkowitz et al. 2005).

### Hydrogen-bonding interactions

To understand intramolecular interactions upon mutations, we have carried out an analysis of H-bonds across the wild and mutant protein molecules, respectively. We employed the Swiss-PDB viewer program to visualize protein complexes and compute H-bonding (Guex 1996; Guex and Peitsch 1996).

## Results

### Identification of nsSNPs and their selection using F-SNP database

The F-SNP database resource (Lee and Shatkay 2008) lists 11 nsSNPs out of the 941 SNPs for CASP9 gene. They are rs1052576, rs1052574, rs2308939, rs2308949, rs2308950, rs1820204, rs2020897, rs2308938, rs2308941, rs9282624 and rs4646008. The corresponding allele change of these nsSNPs is also obtained from F-SNP. SIFT and PolyPhen tools are employed to obtain the corresponding changes in the amino acid residues of these nsSNPs. F-SNP calculates a specific functional significance (FS) score for each of these nsSNPs which signifies their damaging effects (Lee and Shatkay 2008, 2009). FS scores computed by F-SNP database are found to be 0.774, 0.789, 0.977, 0.136, 0.318, 0.888, 0.916, 0.533, 0.749, 0.919 and 0.774 for the 11 nsSNPs rs1052576, rs1052574, rs2308939, rs2308949, rs2308950, rs1820204, rs2020897, rs2308938, rs2308941, rs9282624 and rs4646008, respectively, as depicted in Table 1.

**Table 1:** Lists of nsSNPs identified by F-SNP database with their corresponding alleles and amino acid changes

SNP ID	F-SNP			Amino acid change with position	
	SNP type	Allele change	FS score	SIFT	PolyPhen
rs1052576	Non-synonymous	T^a^/G^b^	0.774	Q^c^(221)R^d^	Q^c^(221)R^d^
rs1052574	Non-synonymous	T^a^/C^e^	0.789	L^f^(197)P^g^	L^f^(197)P^g^
rs2308939	Non-synonymous	C^e^/A^h^	0.977	R^d^(192)S^i^	R^d^(192)S^i^
rs2308949	Non-synonymous	G^b^/A^h^	0.136	G^j^(176)R^d^	G^j^(176)R^d^
rs2308950	Non-synonymous	G^b^/A^h^	0.318	R^d^(173)H^k^	R^d^(173)H^k^
rs1820204	Non-synonymous	T^a^/A^h^	0.888	F^l^(136)L^f^	F^l^(136)L^f^
rs2020897	Non-synonymous	G^b^/C^e^	0.916	E^m^(114)D^n^	E^m^(114)D^n^
rs2308938	Non-synonymous	C^e^/T^a^	0.533	L^f^(106)F^l^	L^f^(106)F^l^
rs2308941	Non-synonymous	C^e^/T^a^	0.749	T^o^(102)I^p^	T^o^(102)I^p^
rs9282624	Non-synonymous	C^e^/G^b^	0.919	I^p^(185)M^q^	I^p^(185)M^q^
rs4646008	Non-synonymous	C^e^/T^a^	0.774	S^i^(99)L^f^	S^i^(99)L^f^

*Fs* Functional significance

^a^Thymine

^b^Guanine

^c^Glutamine

^d^Arginine

^e^Cytosine

^f^Leucine

^g^Proline

^h^Adenine

^i^Serine

^j^Glycine

^k^Histidine

^l^Phenylalanine

^m^Glutamate

^n^Aspartate

^o^Threonine

^p^Isoleucine

^q^Methionine

### Functional prediction of nsSNPs using F-SNP database

F-SNP database predicts the deleterious effect of SNPs with respect to protein coding, splicing regulation, transcriptional regulation and post-translational effects (Lee and Shatkay 2008). To obtain the damaging functional effect of nsSNPs, F-SNP integrates multiple tools that are based on different algorithms and computes the FS score for each of them. The deleterious SNP has FS score value between 0.5 and 1 (Lee and Shatkay 2009). Nine nsSNPs (rs1052576, rs1052574, rs2308939, rs1820204, rs2020897, rs2308938, rs2308941, rs9282624 and rs4646008) are found to have significant FS scores in the range of 0.5–1 as depicted in Table 1.

The nsSNP, rs1052576, with FS score of 0.774 is found to have a deleterious effect on the protein coding region as predicted by SNP-effect tool. Splicing regulation system is found to be altered by ESEfinder. Changes in transcriptional regulatory region and in post-translational modification site are examined by Golden Path and OGPET, respectively. The nsSNP, rs1052574, with FS score of 0.789 is predicted to be damaging by SIFT, PolyPhen, SNPeffect, LS-SNP and SNPs3D tools, which in turn are queried by F-SNP database to predict the functional impact on protein coding region. ESEfinder and ESRSearch predict changes in the splicing regulation system. Golden Path examines a change in transcriptional regulatory region. For nsSNP, rs2308939, with FS score of 0.977, protein coding region is predicted to be deleterious by PolyPhen, SNPeffect and SNPs3D tools. Splicing regulation system, transcriptional regulatory region and post-translational modification site for rs2308939 are examined to have changes by their respective tools (Table 2).

**Table 2:** Functional effect of nsSNPs by F-SNP database

Functional category	Prediction tools	Prediction results of SNPs				
		rs1052576 FS score: 0.774	rs1052574 FS score: 0.789	rs2308939 FS score: 0.977	rs1820204 FS score: 0.888	rs2020897 FS score: 0.916
Protein coding	PolyPhen	Benign	Damaging	Damaging	Damaging	Benign
	SIFT	Tolerated	Damaging	Tolerated	Tolerated	Damaging
	SNP-effect	Deleterious	Deleterious	Deleterious	No entry	Benign
	LS-SNP	Benign	Deleterious	Benign	Benign	Benign
	SNPs3D	Benign	Deleterious	Deleterious	Benign	Deleterious
Splicing	ESEfinder	Changed	Changed	Changed	Changed	Changed
Regulation	ESRSearch	Not changed	Changed	Changed	Changed	Changed
	PESE	Not changed	Not changed	Changed	Changed	Changed
	RESCUE-ESE	Not changed	Not changed	Changed	Changed	Changed
Transcriptional regulation	Golden Path	Exist	Exist	Exist	Exist	Exist
Post-translation	OGPET	Exist	Not exist	Exist	Exist	Not exist
		rs2308938 FS score: 0.533	rs2308941 FS score: 0.749	rs9282624 FS score: 0.919	rs4646008 FS score: 0.774	
Protein coding	PolyPhen	Benign	Damaging	Damaging	Damaging	
	SIFT	Tolerated	Tolerated	Tolerated	Tolerated	
	SNP-effect	Benign	Deleterious	Benign	Deleterious	
	LS-SNP	Benign	Benign	Benign	Benign	
	SNPs3D	Deleterious	Deleterious	Benign	No entry	
Splicing regulation	ESEfinder	Not changed	Changed	Changed	Not changed	
	ESRSearch	Changed	Changed	Changed	Changed	
	PESE	Changed	Not changed	Changed	Not changed	
	RESCUE-ESE	Changed	Not changed	Changed	Changed	
Transcriptional regulation	Golden Path	Exist	Exist	Exist	Exist	
Post-translation	OGPET	Not exist	Exist	Not exist	Exist	

*FS* Functional significance

For nsSNP, rs1820204 with FS score 0.888, PolyPhen predicts a deleterious effect in protein coding region. Splicing regulation system, transcriptional regulatory region and post-translational modification site for rs1820204 are examined to have changes by their respective tools. The nsSNP, rs2020897 with FS score 0.916, protein coding region is predicted to be deleterious by SIFT and SNPs3D. Changes in transcriptional regulatory region and in post-translational modification site are examined by Golden Path and OGPET, respectively. SNPs3D predicts a deleterious effect on protein coding region for rs2308938 with FS score of 0.533. A change in exonic-splice region is predicted by ESRSearch, PESE and RESCUE-ESE. Golden Path examines a change in the transcriptional regulatory region (Table 2).

The nsSNP, rs2308941, with FS score 0.749 is predicted to be deleterious by PolyPhen, SNPeffect and SNPs3D tools. A change exists in exonic-splice region as predicted by ESEfinder and ESRSearch. Changes in transcriptional regulatory region and in post-translational modification site are examined by Golden Path and OGPET, respectively. For nsSNP, rs9282624 with FS score 0.919, PolyPhen predicts a deleterious effect on protein coding region. Changes in splicing regulation system and transcriptional regulatory region are examined by their respective tools. For nsSNP, rs4646008 with FS score 0.774, protein coding region is predicted to be deleterious by PolyPhen and SNP-effect. ESRSearch and RESCUE-ESE predict changes in splicing regulation system. Changes in transcriptional regulatory region and in post-translational modification site are examined by Golden Path and OGPET, respectively (Table 2).

### Phenotypic effect of nsSNPs predicted by nsSNPAnalyzer

The nine nsSNPs (rs1052576, rs1052574, rs2308939, rs1820204, rs2020897, rs2308938, rs2308941, rs9282624 and rs4646008) having significant FS scores are annotated by nsSNPAnalyzer to evaluate their phenotypic class (disease associated vs. neutral) (Bao et al. 2005). The ENVIRONMENT program (Bowie et al. 1991) evaluates the structural environment of the SNP site based on three structural parameters (area buried, fraction polar and secondary structure). Among these nine nsSNPs, rs1052574 is annotated to be in the buried area (B1) with a score of 0.637 and fraction polar score of 0.188. A deleterious SIFT score of 0.00 is also predicted for rs1052574 and hence, this nsSNP is classified to be disease associated by nsSNPAnalyzer. The nsSNP, rs2308939 is classified to be neutral which is in the partially buried category of P2 with area buried score of 0.351, fraction polar score of 0.771 and SIFT score of 0.58. Another nsSNP, rs9282624 is classified to be neutral which is in the partially buried category of P2 with area buried score of 0.401, fraction polar score of 0.760 and SIFT score of 0.07. The other six nsSNPs (rs1052576, rs1820204, rs2020897, rs2308938, rs2308941 and rs4646008) are not evaluated by the ENVIRONMENT program. Hence, rs1052574 is predicted to be functionally important. The phenotypic classes of nsSNPs are depicted in Table 3.

**Table 3:** Phenotypic classes of nsSNPs obtained from nsSNPAnalyzer

SNP ID	Area buried	Fraction polar	Environment	Secondary structure	SIFT score	Phenotype
rs1052574	0.637	0.188	B1^a^	Helix	0.00	Disease
rs2308939	0.351	0.771	P2^b^	Helix	0.58	Neutral
rs9282624	0.401	0.760	P2^b^	Helix	0.07	Neutral

^a^Buried

^b^Partially buried

### Functionally important regions of protein identified by the ConSurf server

To estimate the degree of conservation of the amino acid residues among their close sequence homologs, we have accessed the ConSurf server. Multiple sequence alignment is obtained by CLUSTALW for the query protein sequences with their respective homologous sequences, and a position-specific conservation level score is assigned for each amino acid residue in the alignment using the empirical Bayesian algorithms (Ashkenazy et al. 2010). The amino acid residue leucine at 197 position (L197) in the native protein is found to be conserved with low negative normalized score of −1.048 and a high conservation score of ‘9’. The mutated amino acid residue proline at 197 position (P197) for rs1052574 is also found to be conserved with low negative normalized score of −0.920 and a high conservation score of ‘8’.

### Wild and mutant protein structures obtained from Modeller 9.10

**Fig. 1:**
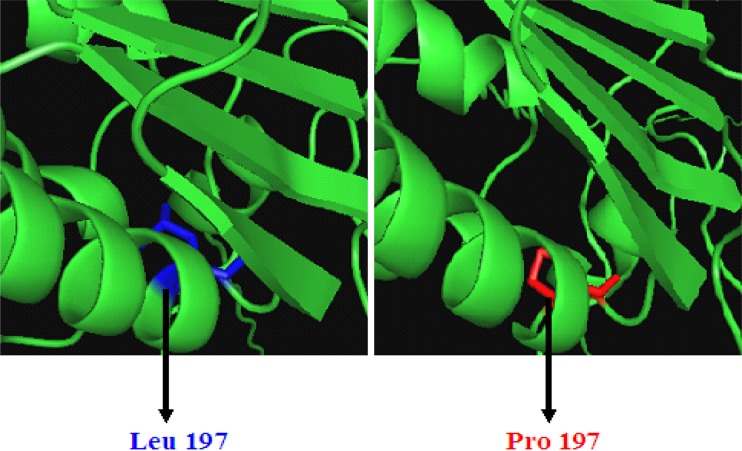
The native and mutant protein structures of SNP rs1052574 where leucine (*Leu*) residue at 197 position in the native protein is replaced by proline (*Pro*) residue in the mutant protein, showing helix to coil transition

The 3D protein structures of both wild and mutant proteins are obtained by comparative homology modeling (Eswar et al. 2007). The program assigns the target sequence and the database of sequences of known structure from PDB as the input to obtain target-template alignments. A better measure of the significance of the alignment is given by the lower expected value (E-value) of the alignment which helps to choose the suitable template candidates (Eswar et al. 2007). The templates (1JXQA, 1NW9A and 2AR9A) having E-value of 0.0 with better sequence coverage with the query protein are chosen for significant sequence alignments. A dendrogram is obtained, which calculates a clustering tree from the input matrix of pairwise distances, to understand the differences among the template candidates (Eswar et al. 2007). The most appropriate template, 1JXQA, with 99 % sequence identity is selected for target-template alignment and construction of the final model. We have shown the native and mutant protein structures of SNP rs1052574 in Fig. 1, where leucine (Leu) residue at 197 position in the native protein is replaced by proline (Pro) residue in the mutant protein, showing helix to coil transition.

### Predicting protein stability changes upon mutation

The empirical protein design force-field FoldX (Guerois et al. 2002) is used by SNPeffect (Reumers et al. 2005) to calculate the difference in free energy of the mutation: delta delta G (ddG). The homology model build for the query protein sequence is evaluated by the FoldX program and found that the mutation from leucine to proline at position 197 results in a ddG of 7.74 kcal/mol.

### Intramolecular H-bonding interactions

**Fig. 2:**
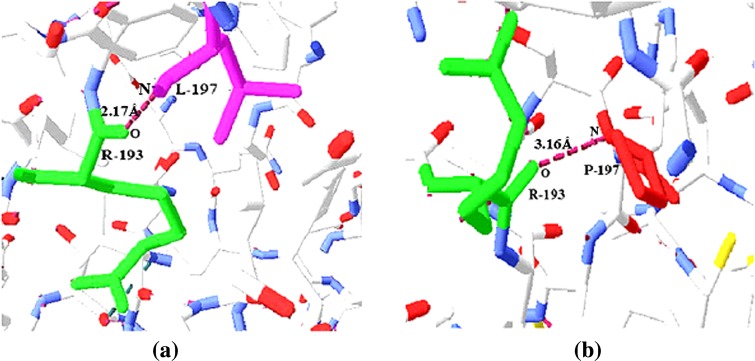
Intramolecular interactions in the native (**a**) and mutant (*L197P*) (**b**) of CASP9 protein models. **a** Hydrogen bond with a distance of 2.17 Å between oxygen (*O*) of arginine (*R193*) and nitrogen (*N*) of leucine (*L197*) in the native CASP9 protein. **b** Hydrogen bond with a distance of 3.16 Å between oxygen (*O*) of arginine (*R193*) and nitrogen (*N*) of proline (*P197*) in the mutant CASP9 protein

The significant nsSNP, rs1052574 of CASP9 producing the amino acid variant L197P shows a helix to coil transition. The effect of intramolecular interactions upon mutation is analyzed by computing H-bonding in the wild and mutant protein molecules. In the native CASP9 protein, leucine197 (L197) interacts with arginine (R193), showing a strong H-bonding with a distance of 2.17 Å between N of L197 and O of R193, as depicted in Fig. 2. Amino acid substitution from L197 to P197 shows a weak H-bonding in the mutant protein with a distance of 3.16 Å between N of P197 and O of R193, as represented in Fig. 2.

## Discussion

We retrieved a total of 941 SNPs associated with CASP9 gene and identified 11 SNPs to be non-synonymous (rs1052576, rs1052574, rs2308939, rs2308949, rs2308950, rs1820204, rs2020897, rs2308938, rs2308941, rs9282624 and rs4646008). Among these, nine nsSNPs (rs1052576, rs1052574, rs2308939, rs1820204, rs2020897, rs2308938, rs2308941, rs9282624 and rs4646008) are found to have significant FS scores in the range of 0.5–1. They are then annotated to identify the disease-associated nsSNPs (Bao et al. 2005). Among them, rs1052574 is annotated to be in the buried area (B1) with a deleterious SIFT score of 0.00 and is classified to be disease associated. If the area of the side chain (*A*) >114 Å^2^, the residue is placed in the environment class B1 when fraction polar (*f*) <0.45. If 0.45 ≤ *f* < 0.58, then the environment class is B2 and the environment class is B3 when *f* ≥ 0.58. If 40 Å^2^ < *A* ≤ 114 Å^2^, the residue is placed in environment category P1 when *f* < 0.67 and environment class P2 when *f* ≥ 0.67. A residue is placed in the exposed environment category E if less than 40 Å^2^ of the side chain is buried (Bowie et al. 1991). SIFT program (Ng and Henikoff 2003) uses an empirical threshold: substitutions with normalized probabilities <0.05 are predicted as deleterious while others are predicted as tolerated. Buried area reflects the solvent accessibility constraint and it is known that disease-associated nsSNPs tend to occur at buried sites (Sunyaev et al. 2000). The SIFT score (Ng and Henikoff 2003) measures the tolerance for a substitution in a multiple sequence alignment and hence incorporates evolutionary information. Thus, rs1052574, residing in the buried site (B1), is predicted to be functionally important. The core residues play a key role in protein folding and stability, and core mutations are considered more deleterious than surface mutations (Cordes and Sauer 1999). The other eight nsSNPs (rs2308939, rs9282624, rs1052576, rs1820204, rs2020897, rs2308938, rs2308941 and rs4646008) are not classified to be disease associated and are not considered in our study.

The significant change in protein coding region is found for rs1052574 which replaces leucine197 in CASP9 protein with proline due to the nucleotide change at 835th position where C**T**G is replaced with C**C**G, i.e., **T** (thymine) to **C** (cytosine). This indicates a transformation of acyclic amino acid to a 5-membered amino acid residue. (The base represented in bold caption is the nsSNP.) Putative ESEs are also predicted for rs1052574 by the change in splicing regulation region. ESEs are short oligonucleotide sequences other than splice sites that enhance splicing from an exonic location. ESEs are recognized by proteins of the SR (serine–arginine) family, which recruit components of the core splicing machinery to nearby splice sites (Fairbrother et al. 2004). Numerous disease associated polymorphisms exert their effects by disrupting the activity of ESEs (Smith et al. 2006). The functionally important regions in these proteins are identified by quantifying the conservation status of amino acid residues among their close sequence homologs (Ashkenazy et al. 2010; Glaser et al. 2003). Both the wild and mutated amino acid residues at position 197 are found to have high conservation score and low (negative) normalized score. Low (negative) normalized scores indicate the conserved positions, while the high scores indicate the variable ones (Goldenberg et al. 2009). If an amino acid in a particular position of a particular protein is conserved, it indicates that this amino acid may be located in an important or functional region of the protein and that its mutation may cause a significant change of the protein’s structure and function (Huang et al. 2010). Comparative study of protein sequences among species infers preliminary information about the protein function, and this has been reported in many genetic diseases. Evolutionarily conserved amino acid residues may serve as a hallmark to identify the functionally critical amino acid of cancer-related genes that may be mutated in tumors (Greenblatt et al. 2003).

Structural information is needed to fully understand the effects and consequences of mutations (Khan and Vihinen 2007). The 3D protein structures of both wild and mutants of CASP9 are obtained by executing comparative homology modeling (Fisher and Sali 2003; Eswar et al. 2007). The L197P substitution for SNP, rs1052574, shows a transition from helix to coil in the modeled protein as shown in Fig. 1. Proline is a very unusual amino acid, in that the side chain cyclizes back on to the backbone amide position. Proline is detrimental to the α-helical conformation for several reasons. First, the amide proton is replaced by a -CH_2_ group, so it is unable to participate in helix stabilization through intramolecular H-bonding. Second, the bulkiness of its pyrrolidine ring places steric constraints on the conformation of the preceding residue in the helix (Williamson 1994; Li et al. 1996). Recent evidence suggests that L166P variant in parkinson protein 7 confers reduced protein stability which may be the underlying cause of early onset Parkinson’s disease (Moore et al. 2003). It is reported that the L166P mutation is located in the α-helix 7 and is predicted to lead to the unfolding of the C-terminal portion of parkinson protein 7 due to the potent helix breaking properties of the substituted proline (Moore et al. 2003). To analyze the structural effect of the nsSNP, rs1052574 on CASP9 protein, we evaluated the energetic impact of the protein upon mutation from leucine to proline at position 197 (Guerois et al. 2002). The mutational free energy change (ddG) is evaluated by FoldX and is found to be 7.74 kcal/mol. The FoldX error margin is around 0.5 kcal/mol. If the mutation destabilizes the structure, ddG is increased, whereas stabilizing mutations decrease the ddG (Schymkowitz et al. 2005). Our result implies that the mutation severely reduces the protein stability. Correct folding and stability are essential for protein function (Gidalevitz et al. 2009). It is also interesting to note that this residue (L197) located in α helix (Fig. 1) resides in the buried region of the native protein (Table 3) and thus, mutation occurring in this residue is critical for protein stability.

The proline substitution may be detrimental to the α-helical conformation by disrupting intramolecular H-bonding (Williamson 1994; Li et al. 1996). Therefore, intramolecular interactions upon mutation are analyzed by computing H-bonds in the native and mutant protein molecules. We have found strong H-bonding in the native protein with a distance of 2.17 Å between L197 and R193, whereas the substitution of L197 to P197 leads to weak H-bonding in the mutant protein with a distance of 3.16 Å between P197 and R193 (Fig. 2). Thus, the substitution of an acyclic amino acid (L197) to a 5-membered amino acid residue (P197), occurring in the buried region of CASP9 protein with weak H-bond interactions, can be critical for the stability in protein monomers. A considerable change in the structure of the protein due to the nsSNP rs1052574 is also observed which shows helix to coil transition in Fig. 1. Hence, due to the change in the structure of the protein and reduction in its stability, the function of the protein may be altered, leading to decreased expression. Protein instability caused due to mutation represents degradation of mutant proteins which can be a prevalent disease-causing mechanism. Degradation of mutant proteins has now been strongly implicated in the etiology of a number of genetic disorders (Waters 2001).

Apoptosis is responsible for the maintenance of homeostasis in tissues as well as in embryonic development. CASP9, belonging to a family of cysteine proteases, is a key regulator of the apoptotic signaling system (Abel et al. 2002). It is an interesting potential tumor suppressor gene in NB, in part due to its localization to human chromosome 1 band p36.1 (Soengas et al. 1999). Thus, the nsSNP, rs1052574, with amino acid variant (L197P) showing a deleterious phenotypic effect and reduced protein stability can decrease the function of the protein which may lead to reduced expression of CASP9. Thereby, it can alter the apoptotic signaling system which can be detrimental and result in the development of NB. Dysregulation of apoptosis is likely to be instrumental in the development of childhood tumor neuroblastoma, and it has been studied that polymorphism in CASP9 gene leads to reduced expression of the protein in NB tumors (Abel et al. 2005; Abel et al. 2002). Mutations in genes encoding proteases, as well as inhibitors and regulators of proteolytic pathways, have been shown to cause numerous human diseases, both inherited and acquired (oncogenic) (Kato 1999; Vu and Sakamoto 2000).

Hence, the results of the nsSNP, rs1052574, for CASP9 gene which is predicted to have a deleterious phenotypic effect and reduced protein stability may be considered to be significant, reducing the function of the apoptotic protease cascade and thereby, leading to the development of neuroblastoma. Our results obtained from these in silico studies, may provide an insight into the genetic mechanism of the disease.

## Electronic supplementary material

Below is the link to the electronic supplementary material.

Supplementary material 1 (DOC 38 kb)
