# Supplementary material for: Leucine to proline substitution by SNP at position 197 in Caspase-9 gene expression leads to neuroblastoma: a bioinformatics analysis
Source: 3 Biotech. 2012 Sep 18;3(3):225–34. doi: 10.1007/s13205-012-0088-y (PMC3646108; doi:10.1007/s13205-012-0088-y)
Supplement: Supplementary file 1 — Supplementary material 1 (DOC 38 kb) [file 13205_2012_88_MOESM1_ESM.doc]

Supplementary Table: SNP data set of CASP9

rs16701156, rs16701155, rs16701154, rs16701153, rs16701152, rs16701151, rs16701150, rs16701149, rs16701148, rs16701147, rs16701146, rs16701145, rs16701144, rs16701143, rs16701142, rs16701141, rs16701140, rs16701139, rs16701138, rs16701137, rs16701136, rs16701135, rs16701133, rs16013795, rs16013792, rs16013789, rs16013787, rs16013786, rs14700481, rs13799690, rs13799689, rs13799688, rs13799687, rs10727060, rs10726901, rs10726874, rs8160342, rs8155283, rs52570359, rs52561239, rs52467427, rs52377637, rs52354230, rs52345501, rs52315095, rs52284457, rs52088619, rs51974270, rs51910822, rs51907741, rs51828161, rs51721990, rs51716988, rs51648636, rs51371854, rs51366753, rs51351655, rs51322405, rs51262415, rs51101109, rs51025398, rs50832424, rs50828662, rs50529350, rs50433317, rs50352972, rs50331380, rs50300866, rs50199329, rs50111416, rs50011822, rs49998972, rs49992103, rs49853342, rs49822454, rs49622092, rs49605848, rs49380558, rs49380160, rs49363993, rs49320418, rs49260707, rs49116227, rs49104043, rs49097566, rs49039921, rs48931460, rs48898539, rs48872072, rs48661512, rs48445713, rs48370606, rs48280980, rs48262129, rs48221459, rs48214926, rs48211701, rs48083528, rs48052409, rs47975247, rs47974392, rs47776595, rs47706467, rs47599897, rs47493111, rs47422441, rs47174412, rs47078424, rs46817238, rs46746812, rs46525560, rs46496308, rs46323025, rs46223381, rs46182064, rs46168256, rs46017382, rs45733961, rs45727024, rs32868021, rs32671662, rs32386517, rs32144129, rs32134344, rs32000006, rs31910318, rs31799586, rs29412528, rs27609434, rs2760943, rs27609432, rs27609431, rs27609430, rs27609429, rs27609428, rs27609427, rs27609426, rs27609425, rs27609424, rs27609423, rs27609422, rs27609421, rs27609420, rs27609419, rs27609418, rs27609417, rs27609416, rs27609415, rs27609414, rs27609413, rs27609412, rs27609411, rs27609410, rs27609409, rs27609408, rs27609407, rs27609406, rs27609405, rs27609404, rs27609403, rs27609402, rs27609401, rs27609400, rs27609399, rs27609398, rs27609397, rs27609396, rs27609395, rs27609394, rs27609393, rs27609392, rs27609391, rs27609390, rs27609389, rs27609388, rs27609387, rs27609386, rs27609385, rs27609384, rs27609383, rs2760938, rs27609381, rs27609380, rs27609379, rs27609378, rs27609377, rs27609376, rs27609375, rs27609374, rs27609373, rs27609372, rs27609371, rs27609370, rs2760936, rs27609368, rs27609367, rs27609366, rs27609365, rs27609364, rs27609363, rs27609362, rs27609361, rs27609360, rs27609359, rs27609358, rs27609357, rs27609356, rs27609355, rs27609354, rs27609353, rs27609352, rs27609351, rs27609350, rs27609349, rs27609348, rs27609347, rs27609346, rs27609345, rs27609344, rs27609343, rs27609342, rs27609341, rs27609340, rs27609339, rs27609338, rs27609337, rs27609336, rs27609335, rs27609334, rs27609333, rs27609332, rs16798065, rs16794754, rs16794753, rs16794752, rs16793006, rs16793004, rs16793003, rs16793002, rs16793001, rs16793000, rs16792999, rs16792998, rs16792997, rs16792996, rs16792995, rs16792994, rs16792993, rs16792992, rs16792991, rs16792990, rs16792989, rs16792988, rs16792987, rs16792986, rs16792985, rs16792984, rs16792983, rs16792982, rs16792981, rs16792980, rs16792979, rs16792978, rs16792977, rs16792976, rs16792975, rs16792974, rs16792973, rs16792972, rs16792971, rs16792970, rs16792969, rs16792968, rs16792967, rs16792966, rs16792965, rs16792964, rs16792963, rs16792962, rs16792961, rs16792960, rs16792959, rs16792958, rs16792957, rs16792956, rs16792955, rs16792954, rs16792953, rs16792937, rs16792936, rs16792935, rs16792916, rs16792915, rs16791624, rs16791623, rs16791622, rs16791621, rs16791620, rs16791619, rs16791618, rs16791617, rs16791616, rs16791615, rs16791614, rs16791613, rs16791612, rs16791611, rs16791610, rs16791609, rs16791608, rs16791607, rs16791606, rs16791605, rs16791604, rs16791603, rs16791602, rs16791601, rs16791600, rs16791599, rs16791598, rs16791597, rs16791596, rs16791595, rs16791594, rs16791593, rs16791592, rs16791591, rs16791590, rs16791589, rs16791588, rs16791587, rs16791586, rs16791585, rs16791584, rs16791583, rs16791582, rs16791581, rs16791580, rs16791579, rs16791578, rs16791577, rs16791576, rs16791575, rs16791574, rs16791573, rs16791572, rs16791571, rs16791570, rs16789882, rs16789881, rs16784577, rs16784576, rs16784575, rs16783081, rs16783080, rs16783079, rs16783078, rs16783077, rs16783076, rs16783075, rs16783074, rs16783073, rs16783072, rs16783071, rs16783070, rs16783069, rs16783068, rs16783067, rs16783066, rs16783065, rs16783064, rs16783063, rs16783062, rs16783061, rs16783060, rs16783059, rs16783058, rs16783057, rs16783056, rs16783055, rs16783054, rs16783053, rs16783052, rs16783051, rs16783050, rs16783049, rs16783048, rs16783047, rs16783046, rs16783045, rs16783044, rs16783043, rs16783042, rs16783041, rs16783040, rs16783039, rs16783038, rs16783037, rs16783036, rs16783035, rs16783034, rs16783033, rs16783032, rs16783031, rs16783030, rs16783029, rs16783028, rs16783027, rs16783026, rs16783025, rs16783024, rs16783023, rs16783022, rs16783021, rs16783020, rs16783019, rs16783017, rs16783016, rs16783015, rs16783014, rs16783013, rs16783012, rs16783011, rs16783010, rs16782791, rs16782790, rs16782789, rs16782788, rs16782787, rs16782786, rs16782785, rs16782784, rs16782783, rs16782782, rs16782781, rs16782780, rs16782779, rs16782778, rs16782777, rs16782776, rs16782772, rs16782771, rs16782770, rs16782769, rs16782768, rs16782767, rs6399662, rs6398541, rs6397953, rs6189937, rs6152132, rs22831690, rs22811688, rs22809128, rs22809113, rs22800492, rs22800490, rs22799094, rs22797237, rs22752144, rs22752142, rs22752139, rs22752137, rs22752136, rs8963799, rs8963798, rs8655865, rs26813938, rs26747914, rs26718910, rs26695340, rs26692926, rs26677858, rs26677123, rs26661436, rs26653081, rs26653059, rs26652600, rs26651020, rs26630559,

Supplementary Table contd.

rs26630278, rs26628307, rs26615966, rs26179070, rs26168504, rs24984959, rs24968396, rs61781045, rs61781044, rs61781043, rs61415341, rs61407576, rs61225523, rs61162934, rs61111545, rs61079693, rs61073168, rs61059155, rs60927622, rs60846015,

rs60762788, rs60740707, rs60398711, rs60068985, rs60018729, rs60007091, rs59877385, rs59674188, rs59575363, rs59530664, rs59278275, rs59278072, rs59242130, rs59225907, rs59142583, rs59027153, rs58999320, rs58917548, rs58276993, rs58272110, rs58236802, rs58170571, rs57971278, rs57792149, rs57491775, rs57447510, rs57261756, rs57260997, rs57097032, rs56950083, rs56809060, rs56744980, rs56706128, rs58194091, rs56104933, rs36005936, rs35997775, rs35940515, rs35925725, rs35898948, rs35859635, rs35830046, rs35708904, rs35665235, rs35596930, rs35556426, rs35550418, rs35454115, rs35448662, rs35328037, rs35312186, rs35215874, rs35002772, rs34870949, rs34797624, rs34738793, rs34667779, rs34648160, rs34645131, rs34588807, rs58978195, rs34567745, rs34565023, rs34562967, rs34522526, rs34494163, rs34493839, rs34368154, rs34278747, rs34260715, rs34131377, rs60670586, rs34094056, rs34043261, rs33999574, rs33948521, rs28865341, rs28457091, rs28451126, rs12759381, rs12759214, rs12758034, rs12757814, rs12744432, rs12742731, rs12742433, rs12091224, rs12087057, rs12063779, rs58929583, rs12060237, rs12047854, rs12047848, rs12045879, rs58864633, rs12026303, rs11589076, rs11587521, rs11580743, rs11539576, rs10927794, rs59714348, rs10927793, rs61310368, rs10803388, rs10803387, rs10754896, rs10754895, rs10754894, rs10754893, rs10754892, rs10754891, rs10754890, rs9727501, rs9700738, rs9429243, rs9282625, rs9282624, rs7544759, rs7533287, rs7528524, rs58834547, rs7521899, rs6702284, rs6695562, rs6692024, rs58063508, rs6686966, rs60821877, rs6686890, rs56988303, rs6685648, rs6685218, rs6670978, rs6659615, rs57503761, rs6429748, rs6429747, rs7364791, rs4661639, rs4661638, rs4661637, rs4661636, rs4661334, rs4646111, rs58207664, rs4646110, rs59163513, rs4646109, rs4646107, rs58531527, rs4646106, rs4646105, rs4646104, rs57686402, rs4646103, rs4646102, rs4646101, rs58844063, rs4646100, rs4646099, rs4646097, rs4646096, rs4646095, rs4646094, rs56538661, rs4646093, rs4646092, rs61142188, rs4646091, rs60113551, rs4646090 rs4646089, rs4646088, rs57718147, rs4646087, rs4646086, rs58137147, rs4646085, rs4646084, rs4646083, rs4646082, rs60418645, rs4646081, rs61527922, rs4646080, rs4646079, rs4646078, rs58813184, rs4646077, rs4646076, rs57922890, rs4646075, rs4646074, rs4646073, rs58591226, rs4646072, rs4646071, rs56637341, rs4646070, rs4646069, rs4646068, rs4646067, rs17407048, rs4646066, rs4646065, rs4646064, rs4646063, rs4646062, rs61677156, rs4646061, rs4646060, rs59289624, rs4646059, rs57065746, rs4646058, rs4646057, rs4646056, rs4646055, rs4646054, rs4646053, rs4646052, rs4646051, rs4646050, rs17407055, rs56546311, rs4646049, rs4646048, rs56528906, rs4646047, rs4646046, rs61588522, rs56464007, rs4646045, rs58777144, rs4646044, rs4646043, rs4646042, rs4646041, rs56994886, rs4646040, rs4646038, rs4646037, rs58753630, rs17232874, rs4646036, rs4646035, rs17823120, rs59951497, rs4646034, rs4646033, rs17823138, rs57594865, rs4646032, rs17465642, rs59068447, rs4646031, rs60771680, rs4646030, rs60028262, rs12567816, rs4646029, rs4646028, rs4646027, rs4646026, rs61392567, rs17823144, rs4646025, rs4646024, rs4646023, rs4646022, rs4646021, rs4646020, rs4646019, rs58027389, rs4646018, rs4646017, rs4646016, rs4646015, rs57903961, rs4646014, rs4646013, rs58835991, rs4646012, rs56433850, rs57799211, rs4646011, rs4646010, rs4646009, rs4646008, rs4646007, rs4646006, rs4646005, rs4646004, rs4646003, rs4646002, rs4646001, rs4646000, rs36022335, rs4645999, rs4645998, rs4645997, rs4645996, rs4645995, rs4645994, rs4645993, rs4645992, rs4645991, rs4645990, rs59972351, rs4645989, rs4645988, rs4645987, rs4645986, rs60736966, rs4645985, rs4645984, rs4645983, rs4645982, rs59739777, rs4233538, rs59425114, rs4233537, rs61304672, rs4233536, rs57162155, rs4233535, rs4233534, rs58578189, rs16851700, rs4233533, rs56493059, rs56672804, rs4233532, rs3980016, rs3820069, rs2901965, rs52820803, rs2308950, rs2308949, rs2308948, rs2308947, rs2308946, rs2308945, rs2308944, rs2308943, rs2308942, rs52824490, rs2308941, rs2308940, rs2308939, rs2308938, rs2308937, rs2308936, rs2266727, rs2234723, rs59163705, rs3980017, rs2234722, rs57785763, rs2042370, rs56473223, rs59526731, rs2042369, rs59044575, rs2042368, rs2266733, rs2020904, rs2266732, rs57008520, rs17823126, rs2020903, rs59630474, rs17823132, rs2266731, rs2020902, rs4646098, rs2266729, rs2020901, rs2266728, rs56881689, rs2020900, rs57643632, rs17406894, rs2266726, rs2020898, rs52804336, rs2266725, rs2020897, rs59042533, rs2266724, rs2020895, rs1862712, rs60178269, rs1862711, rs56894205, rs1862710, rs58502353, rs1820205, rs59440431, rs1820204, rs1800622, rs59233515, rs1800616, rs57933874, rs1800615, rs57893595, rs1800614, rs3187133, rs4646108, rs17287082, rs17411965, rs1129347, rs3193040, rs1052596, rs3193034, rs1052592, rs58780274, rs2266730, rs3192987, rs1052576, rs3192985, rs1052574, rs59045053, rs3192982, rs1052571, rs60923687, rs951225, rs12748160, rs59445846, rs933705, rs57969412, rs12758346, rs933704, rs60304487, rs884363, rs883664, rs15421.
